# Acyclovir-Induced Nephrotoxicity: A Case Report

**DOI:** 10.7759/cureus.89597

**Published:** 2025-08-08

**Authors:** Karim Chami, Khalid K Khaleq, Manal El Goubi, Mohmmed Moussaoui, Karim El Aidaoui

**Affiliations:** 1 Anesthesia and Critical Care, Hôpital Universitaire International Cheikh Khalifa Ibn Zaid/Faculty of Pharmacy, Mohammed VI University of Health Sciences, Casablanca, MAR; 2 Anesthesia and Critical Care, Centre Hospitalo-Universitaire Ibn Rochd/Université Hassan II de Casablanca, Casablanca, MAR; 3 Anesthesia and Critical Care, Hôpital Universitaire International Cheikh Khalifa Ibn Zaid/Faculty of Medicine, Mohammed VI University of Health Sciences, Casablanca, MAR

**Keywords:** acute kidney disease, ‏acyclovir, acyclovir crystals, drug-induced acute renal failure, nephrotoxic drugs

## Abstract

Acyclovir is a widely used antiviral medication known for its potential nephrotoxic effects. These adverse effects may include acute kidney injury (AKI), acute tubulointerstitial nephritis, crystal-induced nephropathy, and, in rare cases, tubular dysfunction. While acyclovir is generally considered safe, nephrotoxicity can occur, particularly when administered at high doses or in dehydrated patients. We present the case of a 20-year-old woman who was previously in good health but developed acute kidney failure 48 hours after initiating intravenous acyclovir at a standard meningeal dose of 15 mg/kg every eight hours for suspected herpetic meningoencephalitis. Upon suspicion of drug-induced nephrotoxicity, acyclovir was discontinued, leading to a rapid improvement in renal function within 24 hours. This case highlights the importance of close monitoring in patients receiving acyclovir, particularly those at an increased risk of nephrotoxicity. To ensure early detection and intervention, it is crucial to perform regular renal function assessments, especially within the first 48 hours of treatment.

## Introduction

Acyclovir is a widely prescribed antiviral medication primarily used to treat herpes simplex virus (HSV) and varicella-zoster virus (VZV) infections. While it is generally considered effective and safe, its use, particularly in hospitalized patients receiving high doses or intravenous formulations, carries a notable risk of nephrotoxicity. Studies report that the incidence of acyclovir-induced nephrotoxicity ranges from 12% to 48% [1], with clinical presentations varying from mild, asymptomatic increases in serum creatinine to more severe complications, such as acute kidney injury (AKI), crystal-induced nephropathy, acute tubulointerstitial nephritis, and, in rare instances, tubular dysfunction [2]. The nephrotoxic effects of acyclovir are largely attributed to its poor solubility in urine, which can result in intratubular crystallization, as well as direct tubular toxicity mediated by its metabolite, acyclovir aldehyde [3].

This report describes the case of a young female patient with no pre-existing renal dysfunction who developed acute kidney failure within 48 hours of receiving a standard-dose intravenous acyclovir regimen for suspected herpetic meningoencephalitis. This case further highlights the rapid onset and reversibility of acyclovir-induced nephrotoxicity and emphasizes the critical need for early recognition and timely intervention. Maintaining adequate hydration, particularly in febrile patients with increased fluid loss, is essential to reduce the risk of nephrotoxicity during acyclovir treatment. Other risk factors, such as pre-existing renal impairment or concomitant nephrotoxic medications, should also be considered before initiating therapy.

## Case presentation

A 20-year-old female patient with no prior medical or surgical history presented to the emergency department with febrile altered consciousness, a generalized tonic-clonic seizure, and postictal confusion. The patient did not require endotracheal intubation during her admission. Her level of consciousness progressively improved after the seizure, without further neurological deterioration.

The fever was managed with intravenous paracetamol (1g intravenous every six hours). The generalized tonic-clonic seizure was treated with a single intravenous dose of diazepam. No recurrence was observed, and no long-term antiepileptic therapy was required. No other potentially nephrotoxic medications were administered during hospitalization. Clinical and biological evaluation (stable diuresis and electrolytes) indicated that the patient was adequately hydrated.

 The patient remained hospitalized for eight days. Her encephalopathy resolved within 72 hours after admission. Cerebrospinal fluid (CSF) analysis revealed a lymphocytic predominance, while brain MRI showed no abnormalities. With the suspicion of herpetic meningoencephalitis, the patient was initiated on intravenous acyclovir at a meningeal dose (15 mg/kg every eight hours). However, 48 hours after starting treatment, she developed a sudden decline in renal function, with creatinine levels increasing from 5.75 mg/L to 47.78 mg/L and urea levels rising from 0.18 mmol/L to 0.73 mmol/L. Despite this deterioration, urine output remained stable at 2L/24h / 1.4 mL/kg/h, and the electrolyte panel showed no significant abnormalities. Additionally, serum complement levels were normal, and specific antibody tests were negative. Suspecting acute kidney injury secondary to antiviral therapy, acyclovir was promptly discontinued. Following withdrawal of the drug, renal function rapidly improved within 24 hours, with creatinine levels decreasing to 14.11 mg/L and urea levels dropping to 0.41 mmol/L.

**Figure 1:**
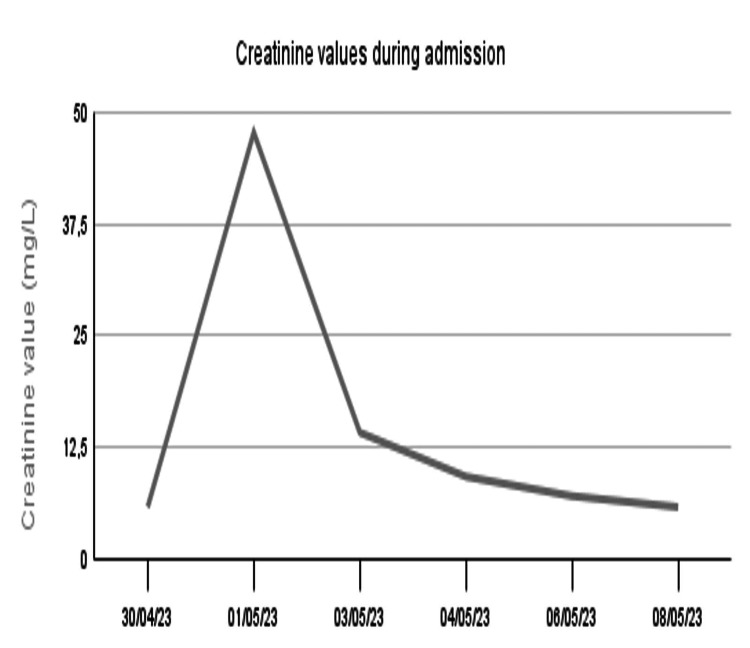
Renal function throughout the hospital stay (Showing when intravenous acyclovir was started and stopped)

Figure 1 shows the timing of acyclovir administration and its relationship to renal function.

## Discussion

This report presents a case of severe acyclovir-induced nephrotoxicity, highlighting the causal relationship between acyclovir administration and its adverse renal effects. The abrupt deterioration in renal parameters within 48 hours of acyclovir administration, followed by rapid improvement after withdrawal, supports a causal relationship. The observed changes in renal biomarkers emerged 48 hours after initiating intravenous acyclovir at meningeal doses, with a rapid improvement in renal function following drug discontinuation. However, blood acyclovir levels were not assessed in this case. This rapid reversibility of kidney injury further supports the diagnosis of acyclovir-induced nephrotoxicity, consistent with previously reported patterns of drug-related AKI. The absence of other nephrotoxic agents and the patient’s stable hemodynamic and electrolyte profile further support acyclovir as the primary cause of renal dysfunction in this case

Acyclovir (C8H11N5O3) is a synthetic guanine nucleoside analogue. Upon entering the body, it undergoes phosphorylation by viral thymidine kinase and host cell enzymes, converting it into its active form, acyclovir triphosphate. This active metabolite competes with guanine triphosphate for integration into viral DNA, leading to chain termination and inhibition of viral replication [4]. The pharmacokinetics of acyclovir are primarily influenced by renal excretion, with approximately 75-80% of the drug eliminated unchanged in the urine. Under normal renal function, its elimination half-life ranges from two to three hours, but this duration can significantly increase in cases of renal impairment, contributing to toxic accumulation [5]. Acyclovir-induced nephrotoxicity is predominantly linked to crystal formation within renal tubules, leading to obstruction and tubular cell damage. Due to its poor solubility in urine, acyclovir has a tendency to crystallize, particularly in patients receiving high doses or those who are dehydrated, resulting in crystalluria. These intratubular crystals can block urine flow and impair glomerular filtration [6]. Beyond crystallization, acyclovir aldehyde, a metabolite of acyclovir, has been implicated in direct renal toxicity. The aldehyde functional group (C=O) within this metabolite is highly reactive, predisposing it to nucleophilic interactions with glutathione, proteins, and nucleic acids. These covalent modifications are thought to contribute to cytotoxicity, particularly in renal tubular cells [7]. In contrast, acyclovir itself lacks significant reactivity and is substantially less toxic. The conversion to acyclovir aldehyde is a critical factor in its nephrotoxic potential, emphasizing the importance of both drug structure and metabolism in renal injury. These mechanisms can result in an acute decline in renal function, as observed in our patient, underscoring the need for early recognition and intervention.

Previous studies have reported variable rates of acyclovir-induced nephrotoxicity, with hospitalization rates for AKI following oral acyclovir/valacyclovir therapy ranging from 0.27% to 10.5%, with a higher risk associated with intravenous administration [8]. Several factors, including high doses, dehydration, and concomitant use of nephrotoxic agents, further elevate the risk of acyclovir-related nephropathy, necessitating careful dose adjustments and adequate hydration. Acute renal failure caused by acyclovir typically develops shortly after treatment initiation, as seen in our case, and is a hallmark of acyclovir-induced nephropathy. This condition is often characterized by preserved urine output, leukocyturia, and crystalluria [9]. The rapid recovery of renal function upon drug discontinuation, as seen in our patient, highlights the reversible nature of this condition. Monitoring renal parameters, such as creatinine and urea levels, is crucial for the early detection of nephrotoxicity and guiding treatment decisions. It is essential to routinely monitor kidney function in patients receiving acyclovir, particularly during the initial days of treatment. Serum creatinine levels should be measured daily or every two days to facilitate early identification of renal impairment. Close monitoring allows for timely intervention and treatment modification if needed. In clinical practice, personalized dosing strategies tailored to patient characteristics and renal function play a key role in reducing the risk of acute renal failure associated with acyclovir therapy. Preventive measures such as maintaining euvolemia, administering acyclovir via slow infusion, and avoiding concurrent nephrotoxic agents are essential for minimizing renal toxicity and optimizing patient outcomes [10].

Future research should focus on developing novel biomarkers for early detection of acyclovir-induced nephrotoxicity, evaluating alternative dosing regimens to reduce renal toxicity, and assessing long-term renal outcomes in patients experiencing acyclovir-related nephropathy. Neutrophil gelatinase-associated lipocalin (NGAL) is a promising serum and urinary biomarker with high sensitivity in detecting acute AKI. Studies indicate that elevated urinary NGAL levels correlate with early tubular injury, preceding detectable renal dysfunction as measured by creatinine [11].

## Conclusions

This case underscores the potential nephrotoxic effects of acyclovir and highlights the importance of proactive monitoring and early intervention to prevent acute renal failure. It underscores the importance of proactive renal function monitoring, especially within the first 48 hours of treatment. Healthcare professionals should be highly attentive to signs of nephrotoxicity, particularly in high-risk patients, and implement a personalized dosing strategy to enhance patient outcomes while reducing the risk of renal complications. Clinicians should ensure adequate hydration, avoid concomitant nephrotoxic drugs, and consider adjusting the infusion rate or dose based on renal function. Although acyclovir is widely used and generally well-tolerated, it can, in some cases, lead to severe nephrotoxicity resulting in acute renal failure. This case emphasizes the critical need for close renal function monitoring during acyclovir therapy, especially in individuals with risk factors for nephrotoxicity. Early recognition and discontinuation of the drug can prevent complications and contribute to a favorable outcome.
